# The Control of the Specificity of CD4 T Cell Responses: Thresholds, Breakpoints, and Ceilings

**DOI:** 10.3389/fimmu.2013.00340

**Published:** 2013-10-23

**Authors:** Andrea J. Sant, Francisco A. Chaves, Scott A. Leddon, Jacqueline Tung

**Affiliations:** ^1^Department of Microbiology and Immunology, David H. Smith Center for Vaccine Biology and Immunology, University of Rochester Medical Center, Rochester, NY, USA

**Keywords:** MHC, CD4 T cells, immunodominance, immunoregulation, HLA-DM

## Abstract

It has been known for over 25 years that CD4 T cell responses are restricted to a finite number of peptide epitopes within pathogens or protein vaccines. These selected peptide epitopes are termed “immunodominant.” Other peptides within the antigen that can bind to host MHC molecules and recruit CD4 T cells as single peptides are termed “cryptic” because they fail to induce responses when expressed in complex proteins or when in competition with other peptides during the immune response. In the last decade, our laboratory has evaluated the mechanisms that underlie the preferential specificity of CD4 T cells and have discovered that both intracellular events within antigen presenting cells, particular selective DM editing, and intercellular regulatory pathways, involving IFN-γ, indoleamine 2,3-dioxygenase, and regulatory T cells, play a role in selecting the final peptide specificity of CD4 T cells. In this review, we summarize our findings, discuss the implications of this work on responses to pathogens and vaccines and speculate on the logic of these regulatory events.

## CD4 T Cell Immunodominance to Foreign Antigens and Pathogens is Peptide Intrinsic and Determined by the Kinetic Stability of Peptide: Class II Complexes

**Figure 1:**
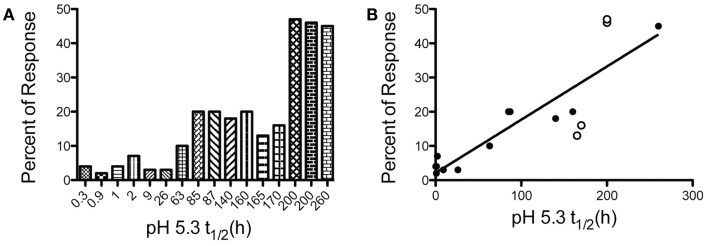
**The kinetic stability of peptide-MHC class II complexes is a key biochemical parameter that dictates CD4 T cell immunodominance**. Test peptide sequences with a range of kinetic stabilities were engrafted into the MalE protein of *E. coli* and used to immunize mice. At the peak of the immune response, CD4 T cells specific for the test peptide or the endogenous MalE epitopes were quantified with IL-2 peptide-specific EliSpot assays. The percent of the total antigen-specific CD4 T cells specific for the test peptides are compared to the spontaneous dissociation rate of peptide-MHC class II complexes at pH 5.3. **(A)** To visualize breakpoints in immunodominance (e.g., cryptic, subdominant, dominant) the magnitude of the peptide-specific response of different peptides are presented in order the increasing kinetic stability of the I-A^d^ peptide complex. **(B)** A near linear relationship is found when between kinetic stability and CD4 T cell immunodominance when values in **(A)** are represented on a linear scale. Filled symbols *R*^2^ = 0.91, with all symbols *R*^2^ = 0.75. Data shown are adapted from previous published studies (5, 8, 9, 36).

There has been tremendous interest in understanding the “rules” of peptide selection by MHC class II molecules and the resulting elicitation of CD4 T cells during the immune response to pathogens or protein vaccines. Many early models supported the importance of proteolytic processing (1, 2) and structural constraints of the peptide within the protein (3, 4) as primary features that determined a peptide’s ability to recruit CD4 T cells. Collectively, these studies suggested that the efficiency of proteolytic release of the peptide was a key determinant of its ultimate immunodominance. However, systematic studies by our laboratory, summarized in Figure 1, on foreign proteins have revealed that the immunodominance of a class II: peptide is due to its intrinsic features, characterized by its spontaneous kinetic stability (5–8). Peptides that successfully recruit CD4 T cells from the endogenous polyclonal T cell repertoire display very slow off-rates (*t*_1/2_ > 75 h at pH 5.3). In contrast, peptides that fail to recruit CD4 T cells dissociate very rapidly from class II molecules (*t*_1/2_ < 10 h). Immunodominance can be manipulated by amino acid changes at sites that anchor the peptide to MHC class II, while leaving T cell receptor (TcR) contact residues within the peptide unperturbed. This latter point is critical, in that with T cell precursor frequency held constant, the kinetic stability of class II peptide complexes behaves as a “rheostat,” up- and down-regulating a peptide’s ability to recruit CD4 T cells. Finally, the finding that a peptide can be moved into a different protein structure, or in different sites on a given protein and maintain its immunodominant or cryptic character (9) has indicated that the property of immunodominance is independent of protein context. This conclusion has important implications for vaccine strategies that seek to incorporate peptides into multi-epitope vaccines [reviewed in Ref. (10, 11)] and suggests that such approaches will allow a peptide to successfully recruit CD4 T cells independently of the context into which it is incorporated and its neighboring peptides.

## The Role of DM Editing in Selecting Immunodominant Epitopes in Response to Protein Vaccination

The MHC-linked DM protein was first discovered because of its role in endosomal release of an invariant chain (Ii) degradation product, a small peptide termed CLIP (Class II invariant chain-derived peptide), that occupies the class II binding pocket immediately after the synthesis of class II and Ii glycoproteins in the endoplasmic reticulum (12). For many allelic forms of class II, this release of CLIP is a requisite event in intracellular peptide loading (13–18). We and others (19–24) showed that DM also has an editing function for removal of endogenous self-peptides from the class II binding pocket, thus shaping the repertoire of “self” recognized by the immune system. Our laboratory showed that for foreign exogenous antigens, DM editing within APC plays a key role in determining immunodominance (5, 7, 19). Highly stable peptide: class II complexes are resistant to DM editing and emerge at the cell surface of APC with high density, while those peptides that bound to MHC class II molecules with low stability are removed by DM during endosomal processing (5–7, 9). These latter types of peptides, cryptic epitopes, thus fail to reach the cell surface at sufficient density to recruit CD4 T cells. The conclusion regarding selective DM editing by our laboratory from *in vivo* and *in vitro* studies and by others [reviewed in Ref. (7, 25–30)] is supported by biochemical and structural studies (31–35, 38) that show that interactions that anchor peptides to class II molecules, particularly at the peptides’s amino terminus, can render these high stability peptide: class II complexes relatively resistant to DM binding and editing.

## Breakpoints and Ceilings of Immunodominance

The direct relationship observed between the kinetic stability of peptide: MHC class II complexes has raised the question of the threshold or breakpoint of immunodominance. If kinetic stability of peptide: class II complexes plays a deterministic role in elicitation of CD4 T cell responses, one can ask, “what is the kinetic stability breakpoint at which it can be certain that a peptide will successfully elicit an immune response?” We have examined this issue most extensively for the murine I-A^d^ molecule in response to intact foreign antigens. Figure 1 indicates the peptide’s off-rate from I-A^d^ at endosomal pH and relative immunodominance when incorporated into the heterologous protein, MalE, the maltose binding protein of *E. coli* (9, 37). This comparison allows internal control for competing peptides within the antigen. From these data (Figure 1A), one can conclude that the breakpoint that allows a cryptic peptide to elicit a readily measurable fraction of the response (e.g., at least 10% of the total response) will have an off-rate (*t*_1/2_) with I-A^d^ in the range of 60–80 h. We have not observed any epitope that fails to recruit CD4 T cells that has a half-life with I-A^d^ >70 h. Similarly, we have not observed any peptide that successfully recruits CD4 T cells that has dissociation half time from I-A^d^ that is <10 h.

In predictions of immunodominance, one issue that is unresolved is what features of the epitope dictates the maximal abundance of CD4 T cells or “ceiling” of the response that any given epitope can achieve. Our studies have revealed that although the requirements for emergence of a peptide into immunodominance are fairly predictable, some peptides show anomalies in the maximal number of CD4 T cells that they recruit, when frequencies are measured at the peak of the response in the local draining lymph node. This phenomenon of variable ceilings is illustrated in Figure 1B, where different antigenic peptides are compared for their ability to recruit CD4 T cells when in MalE. Although there is a linear relationship and positive correlation (filled symbols *R*^2^ = 0.91), between kinetic stability of class II: peptide complexes with immunodominance, some peptides (open symbols) deviate from this relationship. The peptides with somewhat anomalous behavior include Leishmania LACK [161–173] and MalE [69–84], that recruit a larger fraction of the response than predicted from their off-rates from I-A^d^, and others, such as HA [126–138 T > M] that recruit fewer than the expected number of CD4 T cells. We do not yet understand what underlies these differences in maximal response, but can imagine two distinct possibilities. The first is that the DM sensitivity of a given peptide: MHC complex within the priming APC deviates from its spontaneous off-rate, perhaps as a function of the strength of the P1 pocket interaction or the conformation of the complex around this region. For highly DM-resistant peptides, the true initial epitope density displayed by the priming dendritic cells (DC) may thus be increased relative to others with similar spontaneous off-rates from class II. Others may be particularly DM-sensitive and have lower initial epitope density than predicted by their dissociation rate. In agreement with this possibility are the findings of Stern and colleagues (30) who found that DM susceptibility of class II complexes correlates strongly with CD4 T cell recognition and the findings of Mellins and coworkers whose studies revealed disparities between susceptibility to DM editing and the intrinsic off-rates of class II: peptide complexes (38). The second possibility to explain variable ceilings relative to off-rates is that different peptides have TcR contact residues that recruit variable numbers of T cell precursors from the naïve repertoire. The techniques for measuring naïve CD4 T cell precursors specific for single peptide: MHC class II molecules using tetramer-based technology allowed an estimation of number of T cells that recognize individual complexes. These types of empirical approaches (39–41), as well as other theoretical approaches (42) suggest that there can be a significant range in the number of T cells that can respond to different peptide: class II complexes. Such variability has generally been ascribed to negative selection during intrathymic development. It is possible that the final magnitude or ceiling of the response to peptides in the local draining lymph nodes under competitive conditions may be influenced by the precursor frequency of the peptide-reactive CD4 T cells in the host as well as DM editing.

It is important to note that the paradigm we have established between the kinetic stability of peptide: class II complexes, DM editing, and immunogenicity of peptides is expected to hold only for pathogen-derived or foreign peptides that have little homology to “self” proteins in the host. Deletion of CD4 T cells through partial or total homology to self can dramatically re-shape the TcR repertoire, eliminating many of the potentially antigen-reactive CD4 T cells (43–46). There may also be enhanced TcR repertoire selection for some related peptides due to positive selection with peptide analogs (47). Thus, major perturbations of the T cell repertoire for epitopes closely related to “self” is likely to modulate the magnitude of the elicited response and could change classification of some peptides within the categories of immunodominant, subdominant, or cryptic epitopes. Deducing the relative role of DM editing in APC versus precursor frequency in a complex immune response requires more explicit experimentation.

## Competitive CD4 T Cell Responses to Peptide Vaccination

Most of the mechanisms demonstrated or proposed to control immunodominance have centered on the role that endosomal processing and peptide loading onto class II plays in selecting the final specificity of CD4 T cells. It was thus quite surprising to discover in peptide vaccination studies that although cryptic peptides elicit robust immune responses *in vivo* when introduced alone, when they are co-introduced with other peptides, immunodominance hierarchies are established (48). Interestingly, and similar to what we had found for peptides within intact antigens, the immunogenicity of peptides during multi-peptide vaccination correlated with their stability with the presenting class II molecule. Low stability cryptic peptides fail to recruit cells when co-introduced with other, more dominant peptides, while high stability dominant peptides successfully recruit CD4 T cells independently of other peptides that are co-introduced into the host.

There are a number of key features we have discovered in this control CD4 T cell specificity after multi-peptide immunization (48, 49). First, the loss in responses requires that the competing peptides be introduced in the same site, within the same emulsion and most importantly, be presented on the same APC. Thus, the inhibition is local rather than systemic. Second, bystander dominant peptides do not inhibit the response to cryptic peptides through competition for host MHC molecules. Although intuitively appealing, two pieces of data argue this possibility. First, excess exogenously added irrelevant peptides that can bind to host MHC class II molecules do not alter the ability of the agonist cryptic peptide to elicit CD4 T cell responses. Second, dominant peptides presented by unrelated host class II molecules are able to down-regulate responses to co-introduced cryptic low stability peptides (48). Another key aspect of the inhibition is that the termination of expansion occurs midway through the response (at day 4–5). Until this point, there is no detectable influence of the bystander peptides on the response. Finally, there is no evidence that the CD4 T cells specific for dominant peptides render the APC less able to recruit CD4 T cells by “trogocytosing” [reviewed in Ref. (50, 51)] key co-stimulatory or adhesion molecules. Rather, all of the data accumulated thus far suggest that ongoing responses to unrelated dominant peptides down-modulate expansion of CD4 T cells to subdominant peptides through a network of locally active regulatory elements that involve at least IFN-γ, indoleamine 2,3-dioxygenase (IDO), and regulatory T cells (Tregs).

**Figure 2:**
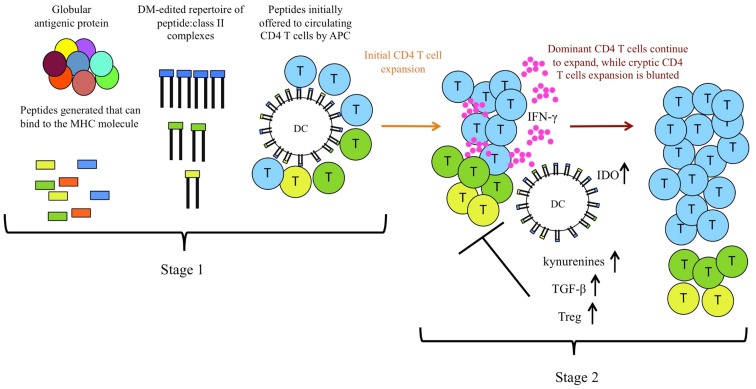
**CD4 T cell immunodominance is shaped by two distinct and selective stages during priming**. In the first stage of selection of CD4 T cells, intracellular DM “editing” promotes immunodominance of high stability class II: peptide complexes by dictating the initial epitope density on priming DC, which allows differential initial recruitment of CD4 T cells. In the second stage, or after multi-peptide immunization, regulatory events induced by dominant CD4 T cells (indicated in blue), including production of IFN-γ, and resulting IDO and Treg induction and production of kynurenines further refine the CD4 repertoire by inhibiting full expansion of CD4 T cells specific for lower stability complexes, indicated in yellow and green.

The components involved in the mechanisms identified are illustrated in Figure 2. All of the data accumulated to date suggested that aborted expansion of CD4 T cells specific for cryptic peptides by responses to high stability dominant peptides is caused by alterations in the local environment during early CD4 T cell expansion and differentiation. Because the expanding populations of CD4 T cells specific for dominant peptides produce IFN-γ as their primary effector cytokine, we tested both IFN-γ-deficient mice and IFN-γR deficient mice and DC, respectively. Use of either genetic model led to significantly diminished negative effects of bystander dominant, CD4 T cell responses (49). IFN-γ is known to have multiple avenues for suppressing CD4 T cell responses [reviewed in Ref. (52–54)]. It can directly induce proliferative arrest and apoptosis cells that bear the IFN-γ-R2 signaling component of the IFN-γ receptor, which is selectively expressed on Th2 cells and naïve CD4 T cells. IFN-γ can also act indirectly to down-modulate adaptive T cell immunity through effects on DC, promoting localized production of IDO. IDO is an immunomodulatory enzyme that has multiple effects on immune responses [reviewed in Ref. (52, 55–60)]. It catalyzes the rate-limiting step in tryptophan degradation, inducing tryptophan deprivation, which can induce the integrated stress response program in T cells (61). Tryptophan degradation also leads to production of tryptophan metabolites, such as kynurenines, that are broadly immunosuppressive. IDO-induced pathways also promote Treg through modification of DC. Several mechanisms have been proposed for the generation of FoxP3-expressing Tregs through IDO. The tryptophan metabolite 3-HAA induces the expression of TGF-β in DC and concurrently causes the conversion of T cells into Tregs (62). Also, Treg generation can be facilitated by binding of kynurenine to the aryl hydrocarbon receptor in T cells (63). These regulatory pathways are critical in counter regulatory immunity to pathogens and in self-tolerance pathways [reviewed in Ref. (55, 64–66)]. We found that inhibition of production of IDO, use of genetic models to eliminate production of, or responsiveness to IFN-γ and finally depletion of Treg, each help rescue suppressed responses to cryptic peptides during multi-peptide immunization. Together, our data suggest that IFN-γ, IDO, and Treg all participate in shaping the specificity of the CD4 T cells elicited during multi-peptide immunization. These regulatory events and mediators also likely participate in the normal contraction of immune responses.

## Summary and Perspectives

Our studies have revealed two distinct mechanisms by which the immune system selects for CD4 T cell reactivity to peptides that persist on the MHC class II molecule, illustrated in Figure 2. Selective DM editing within DC promotes an initial high epitope density of these peptides with class II molecules on the priming APC, while simultaneously removing peptides that cannot sustain interactions with class II. Further regulatory events during competitive responses provide a selective advantage to peptides that persist with class II molecules. The conclusion from this body of work, that there are complementary, reinforcing mechanisms to focus CD4 T cells on peptides which can bind very stably to class II molecules, suggests that persistence *per se* of peptide: class II complexes provide distinct advantages in the adaptive immune response.

There is accumulating evidence that CD4 and CD8 T cells differ in regard to their reliance on continued engagement of their TcR during an immune response. Unlike CD8 T cells that may need only a single encounter with APC to initiate expansion and differentiation (67), CD4 T cells or their progeny appear to require multiple contacts with antigen-bearing APC *in vivo* to expand and differentiate (68). For the delivery of CD4 T cell help to B cells, peptides that can persist on B cells may more effectively recruit follicular helper cells. Even beyond the acute phase of the immune response, CD4 T cells may rely on repeated TcR engagement for some functions (69, 70). Early work on the need for persistent antigen on immunological memory suggested that maintenance of memory CD8 T cells was independent of continued antigen and instead relied heavily on homeostatic proliferation induced by cytokines such as IL-15 [reviewed in Ref. (71)]. CD4 T cells seem to be less well sustained by cytokines alone [reviewed in Ref. (72)] and recent data suggest that low levels of peptide: class II complexes may be critical for maintenance of CD4 T cell memory (73). CD4 T cell dependence on periodic TcR engagement for expansion, differentiation, and memory may underlie the focus of the CD4 T cell response on peptides that bind very stably to the class II molecule. At least during the initial phase of the immune response, even if antigen is eliminated quickly, there will be sufficient complexes on APC to initiate CD4 T cell recognition and then sustain expansion and differentiation. Selective peptide presentation via DM editing, even in the face of diminishing antigen, will promote selective priming of CD4 T cells specific for these complexes, “pruning” the population of CD4 T cells specific for lower affinity ligands. Such early shaping of the CD4 T cell repertoire may allow the early expanding polyclonal responses to be populated by the most desirable of CD4 T cells, thus preventing proliferation of CD4 T cells that later may not be of the most utility in establishing memory or provision of T cell help for B cells. The finding that high stability class II: peptide complexes also favor a diverse CD4 TcR repertoire (74) suggests an additional advantage for these types of complexes in endowing the host with protective immunity. Combined with this early repertoire selection is a second selection event that occurs after peptides are expressed at the APC surface. Here, regulatory events further select CD4 T cells specific for complexes that persist on class II molecules through active suppression of CD4 T cells specific for less “fit” peptide: MHC complexes. These sequential, independent mechanisms likely account for the clear preference of CD4 T cells for high affinity stable peptide: class II complexes.

## Conflict of Interest Statement

The authors declare that the research was conducted in the absence of any commercial or financial relationships that could be construed as a potential conflict of interest.
